# eEF1Bγ binds the Che-1 and TP53 gene promoters and their transcripts

**DOI:** 10.1186/s13046-016-0424-x

**Published:** 2016-09-17

**Authors:** Cinzia Pisani, Annalisa Onori, Francesca Gabanella, Francesca Delle Monache, Antonella Borreca, Martine Ammassari-Teule, Maurizio Fanciulli, Maria Grazia Di Certo, Claudio Passananti, Nicoletta Corbi

**Affiliations:** 1 CNR-Institute of Molecular Biology and Pathology, Department of Molecular Medicine, Sapienza University, Viale Regina Elena 291, 00161 Rome, Italy; 2 CNR -Institute of Cell Biology and Neurobiology, Rome, Italy; 3 IRCCS Fondazione Santa Lucia, Rome, Italy; 4 Department of Research, Advanced Diagnostic, and Technological Innovation, SAFU Laboratory, Regina Elena Cancer Institute, Rome, Italy

**Keywords:** Translation elongation factor, eEF1Bγ, Che-1, AATF, p53, RIP assay, RNA binding protein, Mitochondria, POLR2C, DNA damage

## Abstract

**Background:**

We have previously shown that the eukaryotic elongation factor subunit 1B gamma (eEF1Bγ) interacts with the RNA polymerase II (pol II) alpha-like subunit “C” (POLR2C), alone or complexed, in the pol II enzyme. Moreover, we demonstrated that eEF1Bγ binds the promoter region and the 3’ UTR mRNA of the vimentin gene. These events contribute to localize the vimentin transcript and consequentially its translation, promoting a proper mitochondrial network.

**Methods:**

With the intent of identifying additional transcripts that complex with the eEF1Bγ protein, we performed a series of ribonucleoprotein immunoprecipitation (RIP) assays using a mitochondria-enriched heavy membrane (HM) fraction.

**Results:**

Among the eEF1Bγ complexed transcripts, we found the mRNA encoding the Che-1/AATF multifunctional protein. As reported by other research groups, we found the tumor suppressor p53 transcript complexed with the eEF1Bγ protein. Here, we show for the first time that eEF1Bγ binds not only Che-1 and p53 transcripts but also their promoters. Remarkably, we demonstrate that both the Che-1 transcript and its translated product localize also to the mitochondria and that eEF1Bγ depletion strongly perturbs the mitochondrial network and the correct localization of Che-1. In a doxorubicin (Dox)-induced DNA damage assay we show that eEF1Bγ depletion significantly decreases p53 protein accumulation and slightly impacts on Che-1 accumulation. Importantly, Che-1 and p53 proteins are components of the DNA damage response machinery that maintains genome integrity and prevents tumorigenesis.

**Conclusions:**

Our data support the notion that eEF1Bγ, besides its canonical role in translation, is an RNA-binding protein and a key player in cellular stress responses. We suggest for eEF1Bγ a role as primordial transcription/translation factor that links fundamental steps from transcription control to local translation.

**Electronic supplementary material:**

The online version of this article (doi:10.1186/s13046-016-0424-x) contains supplementary material, which is available to authorized users.

## Background

The eukaryotic elongation factor 1 subunit gamma (eEF1Bγ), also known as the pancreatic tumor-related protein, is a part of the eEF1 multiprotein macromolecular complex. The eEF1 holoenzyme plays a role in protein synthesis by recruiting the aminoacyl-tRNAs to the A site of the ribosome [1]. Using the current nomenclature for higher eukaryotes, eEF1 consists of two different sub-complexes: eEF1A and eEF1B. eEF1A (formerly eEF1α) is a single polypeptide, whereas eEF1B is a multimer of eEF1Bα (formerly eEF1β), eEF1Bδ (formerly eEF1δ), and eEF1Bγ (formerly eEF1γ). There is evidence to indicate that eEF1Bγ stimulates, but is not required for, the catalytic activity of eEF1Bα [2, 3]. Indeed, eEF1Bγ appears dispensable for translation, and its absence does not seem to affect the global rate of translational elongation [4, 5]. Nevertheless, multiple non-canonical roles for eEF1Bγ are emerging, some of which can be regulated by phosphorylation driven by several protein kinases [6]. A role of eEFB1γ in the oxidative stress response pathways is justified by the presence in the N terminus of a conserved sequence resembling the glutathione-binding region of the theta class of glutathione S-transferase (GST) enzymes, which are involved in the detoxification of oxygen radicals. The over-expression of the eEF1Bγ gene product has been reported in several tumors, including pancreatic, breast, colon, and gastric tumors [7–11]. There is growing evidence that the elongation step is also regulated in response to environmental cues, supporting the idea that deregulation of translational control serves as a common mechanism by which diverse oncogenic pathways promote cellular transformation and tumor development [12, 13]. In a wider context, aberrant proliferation of cancer cells is supported by adaptation to nutrient microenvironment mediated by a dynamic metabolic reprogramming [14]. Importantly, the level of eEF1Bγ upregulation was shown to positively correlate with tumor aggressiveness, presumably due to an altered redox balance [2, 15, 16]. eEF1Bγ displays an affinity for membrane and cytoskeleton elements, and it can properly anchor the different subunits of the EF1 complex to the cytoskeleton [2, 6, 17]. Interestingly, Al-Maghrebi et al. (2002) demonstrated the RNA-binding properties of eEF1Bγ by showing for the first time its binding to the 3’ UTR of vimentin mRNA [18], suggesting that eEF1Bγ could exert many of its biological functions through the binding of a pool of mRNAs. In addition, human eEF1Bγ was recently identified in a proteomic screen as a member of the pre-mRNA 3’ end cleavage complex [19]. In this context, eEF1Bγ could participate in the anchoring and translation of a set of mRNAs that are preferentially translated on cytoskeletal- or membrane-bound ribosomes, such as vimentin mRNA. Vimentin has been recently reported to have a regulatory role in supporting the morphology, organization and function of mitochondria [20, 21]. Importantly we previously demonstrated that eEF1Bγ partially co-localizes with mitochondria [5]. Yoo’s research group showed that hCdc73, a component of the human RNA polymerase II-associated factor complex (PAFc), binds and destabilizes p53 mRNA via eEF1Bγ, thus acting as a binding platform [22]. They proposed that mis-regulation of this interaction may lead to tumor progression. Liu et al. reported a new role for eEF1Bγ in the activation of the NF-Kb signaling pathway, through targeting the mitochondrial antiviral adaptor protein (MAVS), which bridges viral RNA recognition and downstream signal activation [23]. The Esposito research group showed that the TNF receptor associated protein (TRAP1), a mitochondrial member of the HSP90 family, which is involved in the protection of oxidative stress, selectively binds eEF1Bγ, and, remarkably, both TRAP1 and eEF1Bγ are co-upregulated in human colorectal cancers [24]. We have previously shown that eEF1Bγ interacts with the RNA polymerase II (pol II) alpha-like subunit “C” (POLR2C), alone or complexed, in pol II [25–27]. The POLR2C/POLR2J heterodimer (also called RPB3/RPB11) is reminiscent of the α subunit homodimer of bacterial RNA polymerase [28]. In bacteria, the alpha subunit homodimer associates with σ factors that mediate promoter recognition [29–31]. Moreover, eEF1Bγ has been described to bind the vimentin 3’ UTR, and we have shown that it also binds the promoter region of the vimentin gene [5, 18]. These results suggest that eEF1Bγ has a role in shuttling/nursing vimentin mRNA (and presumably a specific set of mRNAs) from their gene locus to their appropriate cellular compartment for translation. On the basis of eEF1Bγ sub-cellular localization and its involvement in RNA metabolism and mitochondria/cytoskeleton organization, herein, using a mitochondria-enriched heavy membrane (HM) fraction, we identified, by ribonucleoprotein complex immunoprecipitation (RIP assay), several novel transcripts that complexed with eEF1Bγ. Among the isolated mRNAs, we found genes involved in translation and in mitochondrial/cytoskeleton metabolism. In particular, we found the mRNA of the pol II binding protein Che-1/AATF, and we confirmed the presence of the p53 transcript [22]. Che-1 plays a role in multiple fundamental processes, including control of transcription, cell cycle regulation, DNA damage responses and apoptosis [32–34]. Recent studies suggest that Che-1 protein level dysregulation could be relevant for the transformation process. Che-1 is found upregulated in several leukemia cell lines and in patient with chronic lymphocytic leukemia [33].

Loss or mutation of the oncosuppressor p53 is strongly associated with the susceptibility to cancer and to malignant tumor progression [35, 36]. Notably, Che-1 directly binds p53 and is an important regulator of p53 activity [37]. Moreover, Che-1 enhances the oncogenic potential of the mutated forms of the oncosuppressor p53 (mtp53) [34, 38].

Here, we show for the first time that eEF1Bγ binds to the Che-1 and TP53 promoter regions. In addition, we describe a novel mitochondrial localization for the Che-1 protein, and we show that Che-1 needs mitochondrial integrity for correct localization. We suggest a role for eEF1Bγ as a primordial transcription/translation factor that links the fundamental steps between transcription control and local translation.

## Methods

### Constructs

The myc-tagged pCS2-eEF1Bγ (Myc-eEF1Bγ) (UniProtKB: P26641) construct and its derived deletion mutants were generated by PCR amplification or sub-cloning [5].

The MS2 system vectors were generously provided by Dr. Robert Singer (Albert Einstein College of Medicine, NY). The reporter transcript constructs (Report mRNAs) were obtained by subcloning, in the pMIR-REPORT™-Luciferase plasmid, the 12 repetitions of MS2 RNA stem loop, amplified from pSL1180 vector, and the selected 3’ UTR, amplified from a cDNA library, using the appropriate primers (Additional file 1, Table S1). All constructs were DNA-sequenced by Eurofins Genomics.

### Cell culture and transfections

HeLa human cervical cancer cells and HCT116 human colon carcinoma cells were grown in Dulbecco’s modified Eagle’s medium (DMEM) supplemented with 10 % foetal bovine serum (Gibco-BRL, Grand Island, NY, USA). hSH-SY5Y neuroblastoma cells were grown in DMEM supplemented with 15 % foetal bovine serum. All cell cultures were maintained at 37 °C in a humidified atmosphere of 5 % CO_2_. Transient transfections were performed using Lipofectamine or Lipofectamine 2000 reagents (Thermo Fisher Scientific, Inc., Waltham, MA, USA), according to the manufacturer’s instructions.

The siRNA-mediated interference experiments for eEF1Bγ expression were performed by transfecting SMART pool-specific or non-specific control pool double-stranded RNA oligonucleotides (GE Healthcare Dharmacon Inc., Lafayette, CO, USA) using Lipofectamine 2000.

Drug treatment with doxorubicin (1 μM) (Sigma-Aldrich Co., St. Louis, MO, USA) was carried out by incubating cells with the indicate concentration of the drug in fresh media for either 1 h or 2 h before analysis.

### Sub-cellular fractionation

The mitochondria-enriched heavy membrane (HM) fraction were obtained as previously described [39]. Briefly, HeLa cells (~6 × 10^6^) were harvested in lysis buffer (250 mM sucrose, 20 mM Hepes, pH 7.5, 10 mM KCl, 1.5 mM MgCl_2_, 1 mM EDTA, 1 mM EGTA) and complete protease inhibitor (Roche, Indianapolis, IN, USA). The cells were disrupted by twelve passages through a 25-gauge needle. The HM fractions were obtained by centrifugation at 10,000 *g* for 10 min. The HM pellet was resuspended in high stringency buffer (50 mM Tris–HCl pH 7.4, 250 mM NaCl, 5 mM EDTA, 10 % glycerol, 0.5 % Igepal-CA 630) plus a proteinase inhibitor cocktail (Complete™, Roche, Indianapolis, IN, USA).

The mitochondrial fraction was purified from HeLa and hSH-SY5Y cells (~2 × 10^7^) using a Qproteome Mitochondria Isolation Kit (Qiagen, Hilden, Germany) as previously described [5].

### Immunoblotting

Whole-cell lysate was obtained as previously described, and sub-cellular fractionations (see above) were analyzed by western blotting [5]. The publicly available software ImageJ (National Institutes of Health, USA) was used to quantify the densitometry of the immunoblot bands.

### RIP assay

Mitochondria-enriched heavy membrane (HM) fraction or whole-cell extracts were prepared as above in the presence of RNase inhibitors (Thermo Fisher Scientific, Inc., Waltham, MA, USA). For the immunoprecipitation assay, the protein lysate was pre-cleared for 1 h at 4 °C with Protein A/G-Agarose beads (Roche, Indianapolis, IN, USA) and then immunoprecipitated overnight with the anti-eEF1Bγ rabbit polyclonal antibody or with anti-myc monoclonal antibody. A “no-antibody” immunoprecipitation was performed as a negative control. The beads were washed five times for 5 min at 4 °C with a high stringency buffer and once in PBS buffer. The beads containing the immunoprecipitate samples were collected and resuspended in buffer R (50 mM Tris–HCl pH 7, 10 mM DTT, 5 mM EDTA, 1 % SDS) [40]. A portion of immunoprecipitation was processed for western blot analysis. RNA was extracted using TRIzol® reagent (Thermo Fisher Scientific, Inc., Waltham, MA, USA) according to the manufacturer’s instructions. RNAs were converted to cDNAs and randomly amplified using a Full Spectrum™ Complete Transcriptome RNA Amplification Kit (System Biosciences, Mountain View, CA, USA) according to the manufacturer’s protocol. cDNA was run on a 2 % agarose gel, and the portion between 200 and 500 bp was isolated and cloned into pGEM-T vectors using a pGEM®-T Easy Vector System (Promega, Madison, WI, USA) according to the manufacturer’s instructions. The obtained clones were analyzed by EcoRI digestion, and the selected clones were sequenced by Eurofins MWG Services.

### RNA extraction, retrotranscription and quantitative real-time PCR (qPCR)

Total RNA from HeLa and hSH-SY5Y cells was extracted using TRIzol® reagent according to the manufacturer’s instructions and was then reverse transcribed using a High Capacity cDNA Reverse Transcription kit (Thermo Fisher Scientific, Inc., Waltham, MA, USA). A quantitative real-time PCR (qPCR) assay was performed in triplicate in a 96-well format in an ABI Prism 7000 Sequence Detection System (Applied Biosystems, Foster City, CA, USA) using the SYBR Green PCR Master mix. GAPDH or MT-ND2 was used for the normalization of mRNA, and the relative expression was calculated using the comparative Ct method (2^-ΔΔCt^). Primer sequences used in this study are shown in Additional file 1: Table S1.

### Chromatin immunoprecipitation (ChIP) assay

A chromatin immunoprecipitation assay was performed as previously described [41]. Equal amounts of chromatin from each sample were immunoprecipitated overnight with anti-eEF1Bγ rabbit polyclonal antibodies. The immunoprecipitated sonicated chromatin was amplified using human Che-1-specific primers, human thymidine kinase (TK)-specific primers and human p53-specific primers. The PCR conditions were as follows: 30 cycles at 95 °C for 45 s, 60–67 °C for 30 s, 72 °C for 30 s and a final extension at 72 °C for 5 min.

### Polysome profile analysis

The polysomal profile analysis was performed as previously described [42]. Briefly, cells were homogenized in lysis buffer (10 mM Tris–HCl pH 7.5, 100 mM NaCl, 10 mM MgCl2, 1 % Triton X-100, 30 U/ml RNasin). Lysates were incubated on ice for 5 min and then centrifuged at 12,000 rpm for 5 min at 4 °C. Supernatants were immediately loaded onto a 10 ml 15–50 % (w/v) sucrose gradient and centrifuged at 37,000 rpm for 180 min at 4 °C at in a Beckman SW41 rotor. For EDTA treatments, 100 mM EDTA was added to the cytoplasmic extracts before stratification on a sucrose gradient. Free ribosomal subunits (60S and 40S), monosomes (80S), large polysomes, and the very light mRNPs were detected by UV absorbance at 254 nm using a BioLogic LP system (BioRad Inc., Hercules, CA, USA). Each gradient was collected in 9 fractions, and the proteins were precipitated with a mix containing 50 % ethanol, 25 % methanol and 25 % acetone and were then processed for western blot analysis.

### Immunofluorescence and confocal laser scanning microscopy

Cells were fixed with 4 % formaldehyde in PBS, permeabilized in 0.2 % Igepal-CA 630 (Sigma Chemical Co., St. Louis, MO, USA) for 10 min, and blocked with 1 % BSA in PBS at room temperature. Samples were incubated sequentially with the appropriate primary and secondary antibodies. Slides were mounted with ProLong with DAPI (Thermo Fisher Scientific, Inc., Waltham, MA, USA) or Hoechst 33258 solution (Sigma Chemical Co., St. Louis, MO, USA). To label mitochondria, cells were incubated with 250 nM of MitoTracker® Red CMXRos M7512 (Thermo Fisher Scientific, Inc., Waltham, MA, USA) according to the manufacturer’s instructions and then were fixed and incubated with anti-Che-1 rat polyclonal antibodies. Slides were examined by conventional epifluorescence microscopy (Olympus BX51). Images were captured using a digital camera SPOT RT3 and merged using the IAS2000 software. For confocal laser scanning microscopy, slides were examined with a confocal system TCS-SP5 (Leica Microsystem, GmbH Wetzlar, Germany).

### RNA-FISH combined with immunofluorescence

HeLa cells were processed for immunofluorescence in the presence of RNase inhibitors, with secondary antibody incubation subsequent to FISH to prevent denaturation of the antibodies. FISH was performed using a FITC labeled oligonucleotide probe (Additional file 1: Table S1) [43, 44]. The cells were fixed in 4 % paraformaldehyde in PBS (pH 7.4) for 30 min and washed three times with PBS and 0.2 % Igepal-CA 630 for 5 min. The slides were then permeabilized by treatment with 70 % ethanol overnight at 4 °C. The cells were rehydrated for 5 min in 50 % formamide, 2× SCC (300 mM NaCl, 30 mM sodium citrate, pH 7.0) and pre-hybridized with hybridization buffer (50 % formamide, 10 % dextran sulfate, 2 mM vanadyl-ribonucleoside complex, 40 μg *E. coli* tRNA, 2× SSC) for 1 h at 37 °C. Then, cells were incubated overnight with 30 ng FITC labeled DNA oligonucleotide probe in 40 μl hybridization buffer at 37 °C. The coverslips were washed twice in 2× SCC/50 % formamide at 37 °C and twice in 1× SCC at room temperature. The coverslips were incubated with anti-FITC primary antibodies (to amplify the signal) and the appropriate secondary antibodies in 1 % blocking reagent solution (Roche, Indianapolis, IN, USA) at room temperature and mounted with ProLong with DAPI. The slides were examined by conventional epifluorescence microscopy (Olympus BX51). The images were captured using a digital camera SPOT RT3 and merged using IAS2000 software. Co-localization analysis was performed using Image J software (Image J, Colocalization Coloc 2, Intensity correlation quotient (ICQ)). The ICQ value is calculated based on Li's intensity correlation analysis, which is considered a stable method for co-localization analysis as it allows the discrimination of coincidental events in a heterogeneous situation. ICQ varies from −0.5 (exclusion) to 0.5 (complete co-localization) [45]. Region-of-Interest (ROI) were drawn around single cells. Background and threshold correction were applied for each ROI.

### Antibodies

The following antibodies were used: anti-eEF1Bγ rabbit polyclonal antibody (Bethyl Laboratories, Inc. Montgomery, TX, USA), for western blotting and immunoprecipitation; anti-eEF1Bγ mouse monoclonal antibody (Abnova, Taipei City, Taiwan), for immunofluorescence; anti-myc monoclonal antibody (9E10 clone, hybridoma-conditioned medium), for western blotting and immunoprecipitation; anti-Tom20 rabbit polyclonal antibody (Santa Cruz Biotechnology, Santa Cruz, CA, USA), for western blotting and immunofluorescence; anti-SMN mouse monoclonal antibody (BD Transduction Laboratories, San Jose, CA, USA), for western blotting; anti-L7 rabbit monoclonal antibody (Abcam, Cambridge, UK), for western blotting; anti-S6 rabbit polyclonal antibody (Cell Signaling Technology, Danvers, MA, USA), for western blotting; anti-γ-tubulin monoclonal antibody (Merck Biosciences, Kenilworth, NJ, USA), for western blotting; anti-Che-1 rabbit polyclonal antibody [46], for western blotting; anti-HSP60 monoclonal antibody (Santa Cruz Biotechnology, Santa Cruz, CA, USA), for western blotting; anti β-actin (Sigma-Aldrich Co., St. Louis, MO, USA), for western blotting; anti-FITC mouse monoclonal antibody (Sigma Chemical Co., St. Louis, MO, USA), for RNA-FISH. For Che-1 rat antiserum production, Wistar rats were immunized four times with 250 μg of the purified His tag Che-1 protein every week using Freund’s adjuvants (Difco, Detroit, MI, USA); antiserum was collected 5 days after the last injection. All procedures were carried out in accordance with the ethical guidelines for animal care of the European Community Council (directive 2010/63EU). Housing of the animals meets the behavioral needing of the specie and was supervised by the Responsible Veterinarian. The secondary antibodies conjugated to horseradish peroxidase were purchased from GE Healthcare (GE Healthcare, Chicago, IL, USA). Alexa-Fluor-488 or Alexa-Fluor-594-conjugated secondary antibodies were purchased from Thermo Fisher Scientific (Thermo Fisher Scientific, Inc., Waltham, MA, USA).

## Results

### eEF1Bγ binds specific mRNAs and their gene promoter regions

**Fig. 1:**
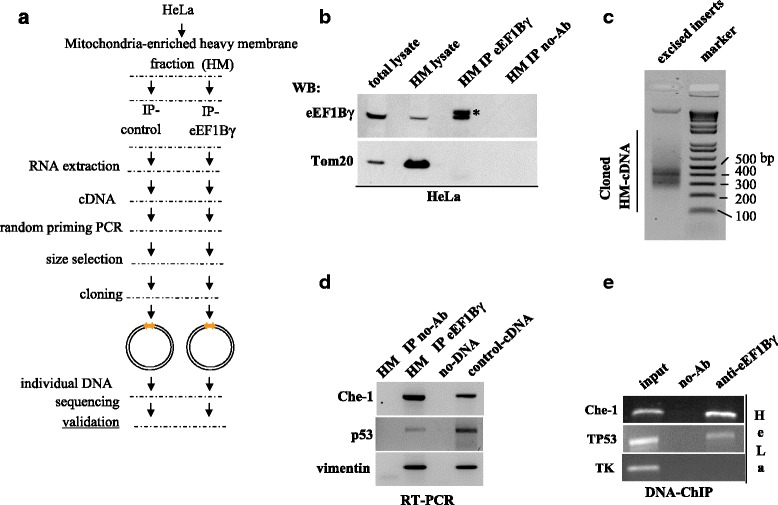
eEF1Bγ binds specific mRNAs and their gene promoter regions **a** Schematic representation of the RIP assay. **b** HeLa cell extract, enriched in heavy membrane (HM) fractions, was immunoprecipitated with anti-eEF1Bγ rabbit polyclonal antibodies or with no antibodies (no-Ab). **c** HM-cDNA library: the RIP assay cDNA output was randomly amplified, size selected and finally cloned. **d** RIP assay output from each sample were analyzed by semi-quantitative RT-PCR performed using primers specific for human Che-1 3’ UTR. Human p53 3’ UTR and human vimentin 3’ UTR were also amplified as positive controls. **e** eEF1Bγ binds to the Che-1 promoter and TP53 promoter at the endogenous chromosomal site. Chromatin immuno-precipitation (ChIP) was performed in HeLa cells using anti-eEF1Bγ rabbit polyclonal antibodies or with no antibodies (no-Ab). Immunoprecipitates from each sample were analyzed by PCR performed with primers specific for the human Che-1 promoter and for the human TP53 promoter. The thymidine kinase human promoter was amplified as a negative control. A sample representing linear amplification of the total input chromatin (input) was included in the PCR as a control

The eEF1Bγ protein has been shown to associate with both the vimentin promoter and vimentin 3' UTR mRNA, thus influencing the cellular shape and mitochondria localization [5, 18]. Here, with the intent of identifying additional transcripts complexed with the eEF1Bγ protein, we performed a series of ribonucleoprotein immunoprecipitation (RIP) assays. A scheme of the RIP assay protocol is shown in Fig. 1a. The HeLa cell mitochondria-enriched HM fraction was immunoprecipitated with anti-eEF1Bγ polyclonal antibodies (Fig. 1b). The co-immunoprecipitated RNA was purified and retro-transcribed. The cDNA output was randomly amplified, size selected and cloned. Fig. 1c shows the resulting HM-cDNA library. Interestingly, among the mRNAs complexed with eEF1Bγ, we isolated the Che-1/AATF mRNA. The RT-PCR analysis, shown in Fig. 1d, demonstrated the presence of Che-1 mRNA in eEF1Bγ RIP assay output.

Although in a specific subcellular fraction, we confirmed the presence of vimentin and p53 transcripts, as previously reported [18, 22]. Additional file 2: Table S2 shows a list of selected individual clones randomly sequenced from the mitochondria-enriched heavy membrane (HM)-cDNA library. Additional file 3: Figure S1A shows the RT-PCR validation of some of the individual clones. The finding of the Che-1 transcript in the eEF1Bγ RIP assay output is consistent with the presence of eEF1Bγ on the Che-1 promoter that we have communicated in our previous manuscript [5]. As shown in Fig. 1e, by DNA ChIP analysis in Hela cells, we confirmed the presence of eEF1Bγ on the Che-1 promoter, and, importantly, we showed for the first time the presence of eEF1Bγ on the TP53 gene promoter. The ChIP experiments were also performed on human neuroblastoma hSH-SY5Y cells, confirming the data (Additional file 4: Figure S2A).

### eEF1Bγ co-localizes with specific mRNAs

**Fig. 2:**
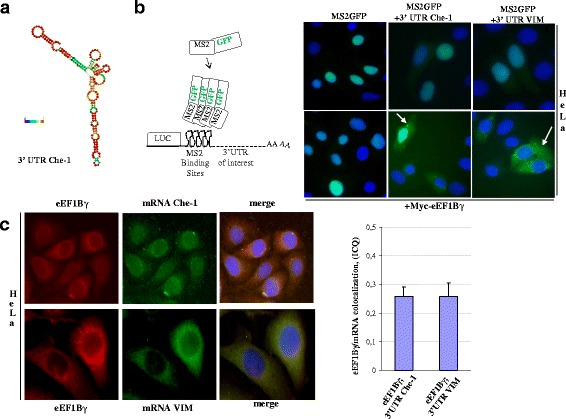
eEF1Bγ co-localizes with specific mRNAs **a** The human Che-1 3′ UTR was folded according to the computer algorithm of Zuker and Stiegler to yield a structure of minimum free energy [65]. **b** Schematic representation of the chimeric bacteriophage MS2 coat protein fused to the GFP protein (MS2-GFP) and the reporter transcript containing multimers of the RNA stem-loop, recognized by the MS2-GFP protein, upstream of the 3’ UTR of the mRNA of interest named: “Report mRNA” (left panel). The MS2-GFP protein was expressed in HeLa cells either alone or with the Report mRNA carrying the 3′ UTR of Che-1 or vimentin mRNAs (upper panel). In the lower panel, MS2-GFP was co-expressed with myc-eEF1Bγ protein and with the Report mRNA carrying either the 3′ UTR of Che-1 or the 3′UTR of vimentin transcripts (right panel). **c** Co-localization of endogenous eEF1Bγ protein and either Che-1 or vimentin endogenous mRNAs in HeLa cells. Expression of eEF1Bγ was detected by indirect immunofluorescence using polyclonal eEF1Bγ antibodies (red), whereas Che-1 and vimentin mRNAs (green) were detected by RNA-FISH. Nuclei (blue) were stained with DAPI. Intensity correlation quotient (ICQ) shown in the bar graph was calculated using Coloc 2 plugin in the Image J/Fiji software and indicates whether the intensity of co-staining varies in synchrony over space. The values indicated represent an average over at least 10 cells from different images and the error bars indicate standard error

It has been shown that in vimentin 3’ UTR mRNA, eEF1Bγ binds an RNA element named the “Y shaped structure”, which exhibits striking sequence homology across species [47, 48]. Moreover, Sasvari et al. demonstrated a role for eEF1Bγ in *Tomato bushy stunt virus* (TBSV) replication by interacting with a stem-loop structure at the 3’ end of the viral RNA [49]. Interestingly, as shown in Fig. 2a, the human Che-1 3′ UTR mRNA is characterized by a stem-loop secondary structure folded according to the dynamic programming algorithm originally proposed by Zuker and Stiegler [50]. To visualize and validate the interaction between eEF1Bγ and the Che-1 3 ’UTR, we performed an MS2 assay [51]. Figure 2b (left panel) shows a schematic representation of the MS2 assay. Briefly, the MS2-GFP system is based on two components: a fusion of the MS2 coat protein with the GFP protein carrying a nuclear localization signal NLS (MS2-GFP) and a reporter transcript (Report mRNA) containing multimers of the RNA stem-loop, recognized by the MS2-GFP protein, upstream of the 3’ UTR of the mRNA of interest. MS2-GFP chimeric protein, over-expressed alone in mammalian cells, shows a nuclear localization, whereas when it binds to the RNA stem-loop, it tends to move in the cytoplasmic compartment. As shown in Fig. 2b (right panel), MS2-GFP protein expressed in HeLa cells alone or with the 3′ UTR of Che-1 or vimentin was analyzed in the presence or absence of myc-eEF1Bγ. Only in the presence of myc-eEF1Bγ a clear dot fluorescent pattern is observed in the cytoplasmic compartment. Additional file 3: Figure S1B shows additional MS2 assays performed on different mRNAs complexed with eEF1Bγ (see Additional file 2: Table S2). As shown in Fig. 2c, to visualize co-localization of endogenous eEF1Bγ protein and endogenous Che-1 or vimentin mRNAs, we performed RNA-FISH analysis combined with indirect eEF1Bγ-immunofluorescence in HeLa cells. We quantified eEF1Bγ/3’UTR mRNA co-localization by measuring intensity correlation quotient (ICQ) values as shown in the bar graph.

### Characterization of the eEF1Bγ mRNA binding property

**Fig. 3:**
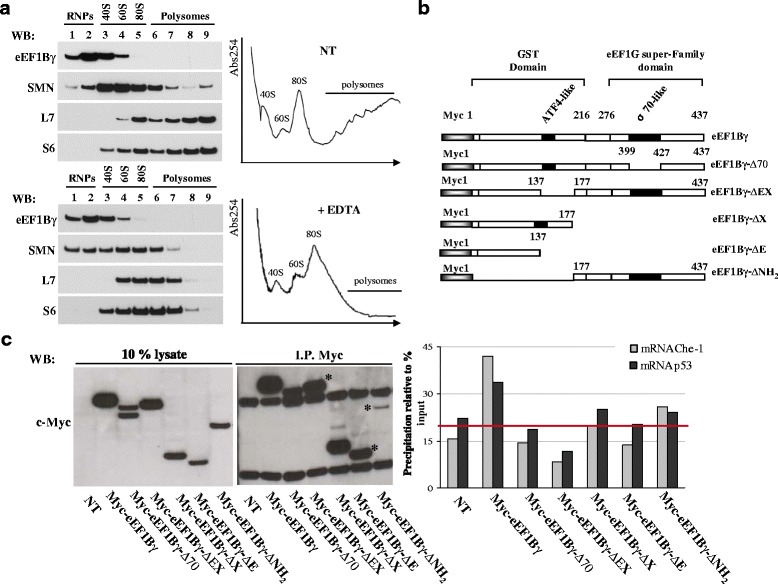
Characterization of the eEF1Bγ mRNA binding property **a** Polysome profiles of HeLa cells by sedimentation velocity through sucrose density gradients. Cytoplasmic extracts were untreated (NT) or treated with 100 mM EDTA to dissociate polyribosomes. Representative absorbance profiles at 254 nm. The 80S monosome peak is indicated (right). Western blot analysis showing the distribution of the eEF1Bγ protein in fractions collected from the top to the bottom of the sucrose gradient. Samples corresponding to mRNPs, 40S, 60S, 80S monosomes and polysomes fractions are indicated. SMN and the ribosomal proteins S6 and L7 were used as controls (left). **b** Schematic representations of myc-tagged full-length eEF1Bγ and its derived deletion mutants transiently transfected in HeLa cells analyzed in panel c. **c** RIP assays were performed with an anti-myc tag antibody. Immunoprecipitated samples were analyzed by western blotting using the myc-tag monoclonal antibodies to verify IP efficiency. The asterisks mark the signal corresponding to the eEF1Bγ mutant, partially covered by the heavy chain Ig band (top) and a non-specific band (bottom). The total cell lysates were immunoblotted to verify the correct expression of the transfected molecules (left). On the right, the graph shows the analysis of mRNAs immunoprecipitated with the indicated constructs. The data are expressed as percent precipitation relative to input mRNAs. The horizontal line illustrates the mean background level

Taking into account that eEF1Bγ acts as an RNA-binding protein, we assessed the eEF1Bγ distribution in a polysomal profile in HeLa cells (Fig. 3a, right). Cytoplasmic extracts were subjected to ultracentrifugation in a sucrose density gradient in the presence or absence of EDTA. The EDTA treatment dissociated the large and small ribosomal subunits and virtually disrupted all polyribosomes. In our experimental conditions, eEF1Bγ protein was mainly retained in slow-sedimenting fractions that were enriched in ribonucleoprotein particles (mRNPs) (Fig. 3a, left). We also tested the distribution of the survival motor neuron (SMN) protein because it has been demonstrated that this RNA-related protein associates with polyribosomes [42, 52]. The protein composition of each collected fraction was validated using S6 and L7 antibodies to monitor the small and large ribosomal subunits, respectively. The eEF1Bγ protein is a multi-domain polypeptide that harbors a GST like domain on the N-terminus and an eEF1G super-family domain at the carboxyl terminus (ref: pFAM 00147). In addition, in the carboxyl terminus, there is a region with 74 % homology to the sigma-70 factors ECF subfamily signature (ref: PDOC00814). To identify the eEF1Bγ domain/s responsible for Che-1 and p53 mRNA interactions, a series of eEF1Bγ deletion mutants fused to the myc tag was constructed (Fig. 3b). HeLa cells were transiently transfected with myc-eEF1Bγ or with its deletion mutants to perform a RIP assay analysis. The immunoprecipitations, performed using myc-tag antibodies, were analyzed by western blotting as shown in Fig. 3c (left panel), and co-immunoprecipitated mRNAs were extracted and converted to cDNA. Che-1 and p53 mRNAs co-immunoprecipitated with the indicated constructs were plotted in a graph. The data are expressed as percent precipitation relative to the input mRNAs. The mean background level is illustrated by the horizontal line in the graph [53]. By RT-PCR, the cDNA output confirmed the presence of both Che-1 and p53 transcripts in full-length eEF1Bγ and their limited presence in deletion mutants eEF1Bγ−ΔNH_2_ and eEF1Bγ−ΔX.

### Che-1 protein mitochondria localization and eEF1Bγ depletion effects

**Fig. 4:**
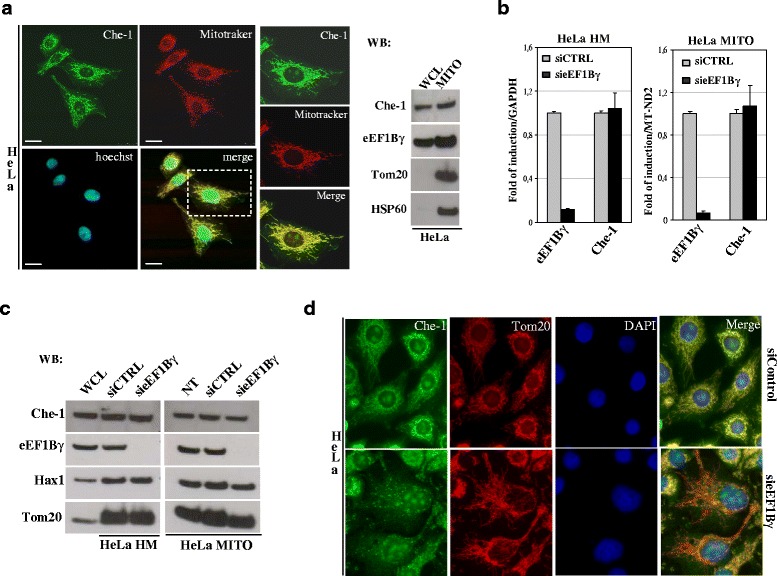
Che-1 mitochondrial localization and impact of eEF1Bγ depletion **a** Co-localization of endogenous Che-1, performed using the anti-Che-1 rat polyclonal antibody (green), and MitoTracker®-Red, which stains mitochondria, in HeLa cells. Extensive co-localization (yellow) between Che-1 and MitoTracker®-Red is visualized by the merged-color image. The panel represents high magnification images of the boxed area. Nuclei were labeled with Hoechst (blue). Scale bars: 10 μm (left panel). On the right western blot analysis of HeLa whole-cell lysate and mitochondrial enriched fractions. The quality of the mitochondrial-enriched fraction was monitored using the anti-Tom20 rabbit polyclonal antibodies and the anti-HSP60 monoclonal antibodies. **b** Quantitative real time RT-PCR (qPCR) analysis of the eEF1Bγ (left) and Che-1 (right) mRNAs in heavy membrane or mitochondrial extracts from HeLa cells (siRNA-Control and siRNA-eEF1Bγ). The gene expression ratio of eEF1Bγ and Che-1, normalized as indicated, are shown as the mean ± SD from three independent experiments performed in triplicate. **c** Che-1 protein levels were determined with a western blot assay in mitochondrial extracts from HeLa cells treated with scrambled siRNA-Control and eEF1Bγ-depleted by specific siRNA. Hax1 and Tom20 were used as mitochondrial markers. **d** Representative fluorescence images of HeLa cells treated with either siRNA-Control or siRNA-eEF1Bγ. Dual-label indirect immunofluorescence was performed with the anti-Che-1 rat polyclonal antibody (green) and the anti-Tom20 rabbit polyclonal antibody (red). Nuclei were stained with DAPI (blue)

We previously demonstrated that eEF1Bγ contributes to govern the correct localization of vimentin intermediate-filament protein, which is known to be involved in cell morphology and organelle positioning [5]. Because the Che-1 protein was observed in both the nucleus and cytoplasmic organelles [33, 54, 55], we examined Che-1 sub cellular localization in more detail. In Fig. 4a (left panel), using the rat Che-1 antibody in a dual-label immunofluorescence assay with mitochondrion-selective dye MitoTracker (red), we detected novel localization of Che-1 protein in mitochondria. To further verify the Che-1 mitochondrial localization, we used the mitochondrial marker Tom20 in a dual-label immunofluorescence assay in hSH-SY5Y cells. Extensive co-localization between endogenous Che-1 and Tom20 is revealed by the merged-color image (Additional file 4: Figure S2B). Western blot analysis of the mitochondria-enriched heavy membrane (HM) fraction, prepared from HeLa cells, clearly confirmed a Che-1 mitochondrial association (Fig. 4a, right panel). Che-1 mitochondrial localization was also observed in the mitochondrial fraction prepared from hSH-SY5Y cells (Additional file 4: Figure S2C). To investigate the possible effects of eEF1Bγ depletion on mitochondrial Che-1 expression levels, quantitative real time PCR (qPCR) and western blot analyses were performed with HeLa cells. In our experimental conditions, eEF1Bγ knockdown did not produce any significant change in Che-1 expression levels (both RNA and protein levels) in mitochondria-enriched HM fractions (Fig. 4b, c). The same results were obtained using a purified mitochondrial fraction (Fig. 4b, c). We also checked transcript and protein levels of Che-1 upon eEF1Bγ depletion by analyzing the whole-cell lysate. Histograms presented in Additional file 3: Figure S1C and S1D show almost no changes in Che-1 levels. Similar results were obtained when qPCR was performed on representative mRNAs co-immunoprecipitated with eEF1Bγ (Additional file 2: Table S2 and Additional file 3: Figure S1C). Equivalent results were obtained for eEF1Bγ siRNA in the hSH-SY5Y cell line (Additional file 4: Figure S2D and E). Next, we investigated the mitochondrial localization of Che-1 by indirect immunofluorescence with both anti-Tom20 antibodies and rat anti-Che-1 antibodies in HeLa cells treated with eEF1Bγ siRNA. Figure 4d shows the results of mitochondrial fragmentation, swelling and disorganization. The mitochondrial network was severely compromised (fragmented) and the co-localization Che-1/Tom20 was partially lost.

### eEF1Bγ in cellular responses to genotoxic stress

**Fig. 5:**
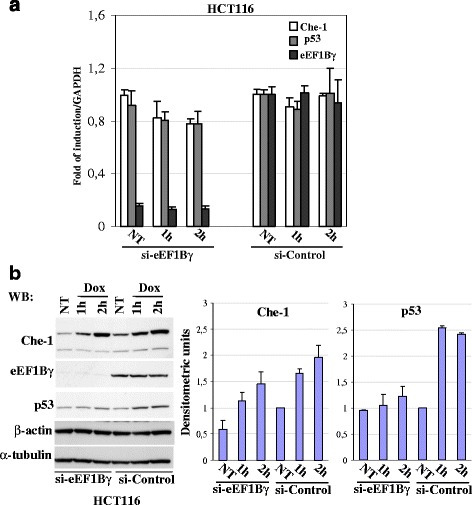
eEF1Bγ depletion: effects on DNA damage response **a** HCT116 cells were transiently transfected with either siRNA-eEF1Bγ or siRNA-Control and 72 h later were treated with 1 μM doxorubicin (Dox) at various time points as indicated. Quantitative real time RT-PCR (qPCR) analysis was performed. The gene expression ratio of eEF1Bγ, Che-1 and p53, normalized as indicated, are shown as the mean ± SD from three independent experiments performed in triplicate. **b** Representative western blot of HCT116 cells (siRNA-Control and siRNA-eEF1Bγ) treated with 1 μM Dox at various time points as indicated. The antibodies that were used are indicated. Densitometric analysis represents the mean ± S.D. of four independent experiments (right panel)

Because our data on eEF1Bγ depletion indicated almost no changes in the Che-1 and p53 levels, we investigated the possible impact of eEF1Bγ in stress pathways shared by Che-1 and p53, such as genotoxic stress induced by treatment with doxorubicin (Dox). To this end, we examined the effect of eEF1Bγ depletion on Che-1 and p53 mRNA and protein levels in HCT116 cells during Dox-induced DNA damage. As shown in Fig. 5a, quantitative real time PCR (qPCR) analysis indicated that HCT116 cells transiently transfected with either siRNA-eEF1Bγ or siRNA-Control and treated with 1 μM Dox at one hour and two hours did not display significant changes of both p53 and Che-1 mRNA levels. Only a slightly decrease of Che-1 and p53 transcripts was detected when Dox treatment was coupled with eEF1Bγ depletion. Western blot analysis indicated that HCT116 cells transiently transfected with either siRNA-eEF1Bγ or siRNA-Control and treated with 1 μM Dox at one hour and two hours produced an evident decrease of p53 protein accumulation and a slight decrease of Che-1 protein accumulation (Fig. 5b) [33, 56].

## Discussion

It is clear that eEF1Bγ, in addition to its canonical role in the translation elongation complex, displays RNA binding ability [18, 22, 48]. In particular, eEF1Bγ has been shown to bind the vimentin 3’ UTR, and we have shown that it also binds the promoter region of the vimentin gene [5, 18]. Here, to identify further functional pathways in which eEF1Bγ is involved, we put our efforts in the isolation and characterization of additional mRNAs recognized by eEF1Bγ protein, using the RIP assay technology. To this end, we focused on the mitochondria-enriched heavy membrane (HM) subcellular fraction with the idea of assessing eEF1Bγ involvement in mitochondrial and cytoskeletal metabolisms. Among the isolated mRNAs, we mainly found genes involved in cytoskeleton transport/organization, translation and mitochondrial metabolism. We confirmed the presence of vimentin and p53 transcripts, already reported [18, 22]. By serendipity, we found the mRNA of the pol-II binding protein Che-1/AATF. The human Che-1 3′UTR is characterized by the presence of a conserved RNA stem-loop structure that could be the target of eEF1Bγ protein. By use of different imaging techniques (MS2-GFP and FISH combined with immunofluorescence), we visualized the co-localization/interaction of endogenous eEF1Bγ with endogenous Che-1 or vimentin mRNAs in peculiar granules accumulated in the cytoplasm around the nucleus.

The polysomal profile analysis reveals that the eEF1Bγ protein is mainly present in the ribosome free mRNPs-enriched fractions. These data together support the notion that the ability of eEF1Bγ to bind selected mRNAs is fundamental to carrying out its non-canonical roles. A further eEF1Bγ non-canonical role resides in its ability to recognize specific gene promoters. We have previously shown that eEF1Bγ binds the promoter region of the vimentin gene, and here, we showed that eEF1Bγ is also found in both Che-1 and TP53 promoters regions. These results suggest a role for eEF1Bγ in nursing/trafficking selected mRNAs from the gene locus to the local product translation site.

These findings are consistent with the following notions: 1) eEF1Bγ binds the p53 transcript and controls its stability, and we show here that eEF1Bγ also binds Che-1 mRNA; 2) Fanciulli and colleagues demonstrated that Che-1 directly interacts with p53 and is involved in regulating p53 expression [37]; and 3) both Che-1 and eEF1Bγ directly bind to the alpha-like pol II heterodimer. More precisely, eEF1Bγ binds to the subunit POLR2C (RPB3), whereas Che-1 contacts the small subunit POLR2J (RPB11). These two subunits form a core subassembly unit of pol II and are considered the functional counterpart of the bacterial RNA polymerase alpha subunit homodimer. In bacteria, the alpha subunit homodimer associate with σ factors that mediate promoter recognition [29–31].

With the aim of characterizing the eEF1Bγ protein domain responsible for mRNA binding, we used a series of eEF1Bγ deletion mutants in RIP assay. In our assays, only two deletion mutants retained minimal Che-1 and p53 mRNA binding ability, thus suggesting that eEF1Bγ protein integrity is required for proper RNA binding activity.

Data reported in the literature have indicated a very wide Che-1 protein distribution, including the nucleolus, nucleus, cytoplasm, Golgi apparatus, centrosome and focal adhesion [33, 54, 55]. We have shown for the first time that Che-1 localizes at the mitochondria. Indeed, Che-1 has been reported to ameliorate mitochondrial dysfunction associated with the accumulation of superoxide [57]. Is it possible that eEF1Bγ is important for transportation of the Che-1 mRNA to the mitochondria, and once the mRNA is there, eEF1Bγ is dispensable for its translation. Garg’s research group recently demonstrated that Che-1 cooperates with miR-2909 in the regulation of mitochondrial uncoupling protein 2 (UCP2), a critical protein whose dysregulation is involved in the pathogenesis of a number of human diseases, including cancer [58, 59]. The connection between eEF1Bγ, Che-1 and p53 proteins and their transcripts indicated, for these genes, involvement in closely related pathways. Indeed, p53 is involved in regulation of the mitochondrial metabolism, playing multiple roles depending on its wild-type/mutation status and translocation into the mitochondria [60–62]. As wells as for Che-1, eEF1Bγ could also participate to p53 localization and/or translation. In this scenario, eEF1Bγ affecting Che-1 and p53 RNA metabolism, could be an important player within functional networks interconnecting Che-1 and p53 proteins. The depletion of eEF1Bγ induces mitochondrial fragmentation and disorganization; this phenomenon correlates with an aberrant Che-1 protein sub-cellular distribution, as we already described for vimentin intermediate filaments [5]. Because in a steady-state condition eEF1Bγ depletion produces almost no changes in Che-1 levels, we investigated the possible impact of eEF1Bγ in stress pathways in which both Che-1 and p53 are involved such as DNA damage [33, 63, 64]. In a Dox-induced genotoxic stress in HCT116 cells, eEF1Bγ depletion decreases p53 protein accumulation and slightly impacts also on Che-1 accumulation. Importantly, Che-1 and p53 proteins are effectors of the DNA damage response machinery that is responsible for maintaining genome integrity and preventing tumorigenesis. Our data are in agreement with the role of eEF1Bγ in cellular stress responses, suggesting that the DNA damage response pathway will be fundamental in further investigations of non-canonical eEF1Bγ functions, pointing also at elucidating eEF1Bγ role in tumorigenesis and cancer progression.

## Conclusions

Using the RIP assay with eEF1Bγ in the mitochondria-enriched HM fraction, we isolated several novel mRNAs involved in cytoskeleton transport/organization, translation and mitochondrial metabolism. Among the eEF1Bγ complexed transcripts, we found the mRNA that encodes Che-1 protein and we confirmed the presence of the p53 transcript. Importantly, we demonstrated that eEF1Bγ binds to both Che-1 and TP53 gene promoters. We described for the first time Che-1 mitochondrial localization. In a Dox-induced DNA damage assay, we show that eEF1Bγ depletion significantly decreases p53 protein accumulation and slightly impacts also on Che-1 protein accumulation. Taking into account that eEF1Bγ is able: 1) to bind directly pol II, 2) to bind to target gene promoters, 3) to bind their transcripts, 4) to accompany the mRNAs to the correct translation site and 5) to participate/enhance translation elongation through its detoxification GST-domain and through its ability to anchor cytoskeleton, we suggest for eEF1Bγ a role in cellular stress responses as primordial transcription/translation factor that links fundamental steps from transcription control to local translation.

## Additional files

Additional file 1: Table S1. Oligos used in the present study. (DOC 56 kb) Additional file 2: Table S2. Different mRNAs associated to eEF1Bγ. (DOC 42 kb) Additional file 3: Figure S1.
**A**. RIP assay output was analyzed by semi-quantitative RT-PCR with specific primers to validate some of mRNAs co-immunoprecipitated with eEF1Bγ and they are listed in Table S2. **B**. The myc-eEF1Bγ and MS2-GFP fusion proteins were expressed in HeLa cells with the report mRNA carrying both the MS2 binding site and the indicated 3′ UTR. **C**. RIP assay-eEF1Bγ mRNAs (Additional file 2: Table S2) analyzed by quantitative real time PCR (qPCR) in HeLa whole-cell lysates treated with siRNA as shown. The gene expression ratio between mRNAs and GAPDH are shown as the mean ± SD from three independent experiments performed in triplicate. **D**. Representative western blot of HeLa whole-cell lysates treated or un-treated with siRNA as shown. The antibodies that were used are indicated. Densitometric analysis represents the mean ± S.D. of 3 independent experiments (right panel). (PDF 208 kb) Additional file 4: Figure S2.
**A**. Chromatin immunoprecipitation was performed in hSH-SY5Y cells using anti-eEF1Bγ rabbit polyclonal antibodies or no-Ab as a control. Immunoprecipitates from each sample were analyzed by PCR performed with primers specific for the human Che-1 promoter and for the human TP53 promoter. The thymidine kinase human promoter was amplified as a negative control. A sample representing linear amplification of the total input chromatin (input) was included in the PCR as a control. **B**. Co-localization of endogenous Che-1, performed with the anti-Che-1 rat polyclonal antibody (green), and the mitochondrial marker Tom20 (red), in hSH-SY5Y cells. Extensive co-localization (yellow) between Che-1 and Tom20 is visualized by the merged-color image. The boxed area represents a high magnification image of co-localization. Nuclei were labeled with DAPI (blue). Scale bars: 10 μm. **C**. Western blot analysis of hSH-SY5Y whole-cell lysate and mitochondrial enriched fraction. The quality of mitochondrial-enriched fractions was monitored using anti-HSP60 monoclonal antibodies and anti-Tom20 rabbit polyclonal antibodies. **D**. Quantitative real time PCR (qPCR) analysis of the eEF1Bγ and Che-1 mRNAs in hSH-SY5Y cells (siRNA-Control and siRNA-eEF1Bγ). The gene expression ratio between eEF1Bγ and GAPDH and between Che-1 and GAPDH are shown as the mean ± SD from three independent experiments performed in triplicate. **E**. Representative Western blot of hSH-SY5Y whole-cell lysates treated or un-treated with siRNA as shown. The antibodies that were used are indicated. (PDF 199 kb)

## Abbreviations

Dox: Doxorubicin
eEF1Bγ: Eukaryotic Elongation Factor subunit 1B gamma
HM: Heavy membrane
MIT: Mitochondria
pol II: RNA polymerase II
POLR2C: RNA polymerase II (pol II) alpha-like subunit “C”
RIP assay: Ribonucleoprotein (RNP) immunoprecipitation assay
