# Supplementary material for: eEF1Bγ binds the Che-1 and TP53 gene promoters and their transcripts
Source: J Exp Clin Cancer Res. 2016 Sep 17;35:146. doi: 10.1186/s13046-016-0424-x (PMC5027090; doi:10.1186/s13046-016-0424-x)
Supplement: Additional file 1: Table S1. — Oligos used in the present study. (DOC 56 kb) [file 13046_2016_424_MOESM1_ESM.doc]

| Primer name | Primer sequence (5’-3’) |
| --- | --- |
| MS2 12 loops F | GGACTAGTTAACAACCGACACTCCTACAAGAA |
| MS2 12 loops R | GGACTAGTGAGCTCACGCGTTAACAACCGACACTCCTACAAGAA |
| 3’UTR-VIM F | GGACTAGTCGAAACTTCTCAGCATCACGATGACCTTG |
| 3’UTR-VIM R | ACGACGCGTGCAGAACCAAGTTGGTTGGATACTTGCTGG |
| 3’UTR-Che-1 F | GGACTAGTACCTCCGACACCCAGTGGGCGCCT |
| 3’UTR-Che-1 R | ACGACGCGTAAGGCGGGGTTGGGCGGGACAGCG |
| 3’UTR-RPS3A F | GGACTAGTCGCAACAATCAGATACGGAAGACC |
| 3’UTR-RPS3A R | ACGACGCGTTGCCACTATTTGAAGTCTGAACTT |
| 3’UTR-Nup160 F | GGACTAGTACAGGAACCCTGTACTCAAGGCCG |
| 3’UTR-Nup160 R | ACGACGCGTGGCTTCTGAAAAACTCAAGAAGGGT |
| 3’UTR-SNX5 F | GGACTAGTGAATAACTGATATGCCTTCACTCA |
| 3’UTR-SNX5 R | ACGACGCGTTCCTAATGTAGATCTGGCCATCTT |
| RT-PCR Che-1 F | GACACCCAGTGGGCGCCTTG |
| RT-PCR Che-1 R | TTAAAGGCGGGGTTGGGCG |
| RT-PCR p53 F | ATGGAGGAGCCGCAGTCAGA |
| RT-PCR p53 R | AGAAGCCCAGACGGAAACCG |
| RT-PCR VIM F | ACCAGCTAACCAACGACAAAG |
| RT-PCR VIM R | GCAGGGTGTTTTCGGCTTC |
| RT-PCR SNX5 F | AAGTGCAAGTGATGCTTGGC |
| RT-PCR SNX5 R | GCCAGGAACAATGTCTCCCTT |
| RT-PCR Nup160 F | TGCTTGGCTATTGTGACCCA |
| RT-PCR Nup160 R | GGAGATCGTGTCTACCCCCA |
| RT-PCR SARS F | GGAGGTCACCGATGCTTGAA |
| RT-PCR SARS R | CATGCGAGGAGACAGGAACA |
| RT-PCR RPS3A F | GGGACGAGACAGGTGCTAAA |
| RT-PCR RPS3A R | AAGCAGTGGTATCAACGCAGA |
| RT-PCR SLC1A4 F | AGCATTTCTGAGCAGGGCTT |
| RT-PCR SLC1A4 R | GGTCTGGGAGTCACAGCAAA |
| RT-PCR GAPDH F | CATGAGAAGTATGACAACAGCCT |
| RT-PCR GAPDH R | AGTCCTTCCACGATACCAAAGT |
| RT-PCR MT-ND2 F | CTACCGCATTCCTACTACTCAACTT |
| RT-PCR MT-ND2 R | GGTGGATGGAATTAAGGGTGT |
| hChe-1 promoter F | CGCGCGCATCGCAATCGCATC |
| hChe-1 promoter R | CGTCACTGCGGGCGTTGCTAG |
| hp53 promoter F | GCAGGAGAATGGCTTGAACC |
| hp53 promoter R | CCAACACCATGCCAGTGTCT |
| hTK promoter F | GCCCCTTTAAACTTGGTGGGCGG |
| hTK promoter R | TTGCGCCTCCGGGAAGTTCACG |
| FLUOR Che-1 | GTAACAATGCTCTTTATTTGTGGCGTTTAAAGGCGGGGTTGGGCGG |
| FLUOR VIM | GGTTGTTAAGAACTAGAGCTTATTCCTATTCCAAATCTATCTTGCGCTCC |

**Additional file1**

Table 1S

Oligos used in the present study.

Sequence of primers and probes are indicated. F indicates forward primer/probe; R stands for reverse primer/probe;
