# Supplementary material for: eEF1Bγ binds the Che-1 and TP53 gene promoters and their transcripts
Source: J Exp Clin Cancer Res. 2016 Sep 17;35:146. doi: 10.1186/s13046-016-0424-x (PMC5027090; doi:10.1186/s13046-016-0424-x)
Supplement: Additional file 2: Table S2. — Different mRNAs associated to eEF1Bγ. (DOC 42 kb) [file 13046_2016_424_MOESM2_ESM.doc]

**Additional file 2**

Table 2S

Different mRNAs associated to eEF1Bγ

| **Gene symbol** | **HGNC ID** | **Name** | **Synonyms** |
| --- | --- | --- | --- |
| Che-1 | 19235 | apoptosis antagonizing transcription factor | AATF,BFR2, DED, TRAUB |
| TP53 | 11998 | tumor protein p53 |  |
| VIM | 12692 | vimentin |  |
| NUP160 | 18017 | nucleoporin 160kDa | FLJ22583, KIAA0197 |
| RPS3A | 10421 | ribosomal protein S3A | S3A |
| RPS15 | 10388 | ribosomal protein S15 | 40S ribosomal protein S15, homolog of rat insulinoma, insulinoma protein, MGC111130, RIG, S15 |
| SNX5 | 14969 | sorting nexin 5 |  |
| SARS | 10537 | seryl-tRNA synthetase | serine tRNA ligase 1, cytoplasmic, SERS |
| TM2D2 | 24127 | TM2 domain containing 2 | BLP1 |
| CAPZA1 | 1488 | capping actin protein of muscle Z-line alpha subunit 1 |  |
| ZNF131 | 12915 | zinc finger protein 131 | pHZ-10, ZBTB35, "zinc finger and BTB domain containing 35 |
| SLC25A51 | 23323 | solute carrier family 25 member 51 | MCART1, mitochondrial carrier triple repeat 1, solute carrier family 25, member 51 |
| SLC1A4 | 10942 | solute carrier family 1 member 4 | alanine/serine/cysteine/threonine transporter, ASCT1, SATT |
| SLC35F2 | 23615 | solute carrier family 35 member F2 | FLJ13018 |
| POR | 9208 | cytochrome p450 oxidoreductase | CYPOR, FLJ26468 |
| SIX4 | 10890 | SIX homeobox 4 | AREC3 |

The table 2S list some individual cDNA clones isolated in RIP assay in complex with eEF1Bγ. Gene name and Gene Nomenclature Committee Identity code (HGNC ID) are reported.
