# Supplementary figures and images for: eEF1Bγ binds the Che-1 and TP53 gene promoters and their transcripts
Source: J Exp Clin Cancer Res. 2016 Sep 17;35:146. doi: 10.1186/s13046-016-0424-x (PMC5027090; doi:10.1186/s13046-016-0424-x)

**A**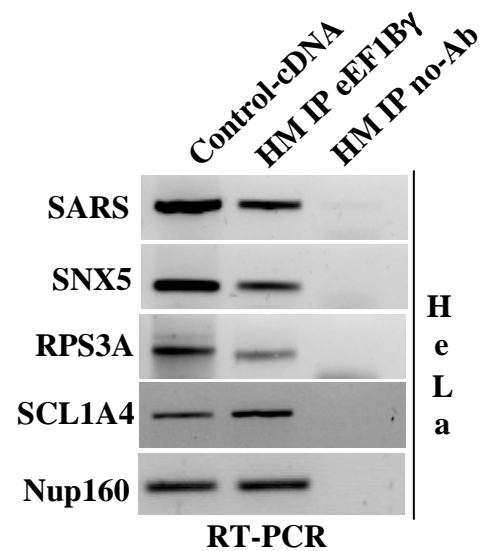**B**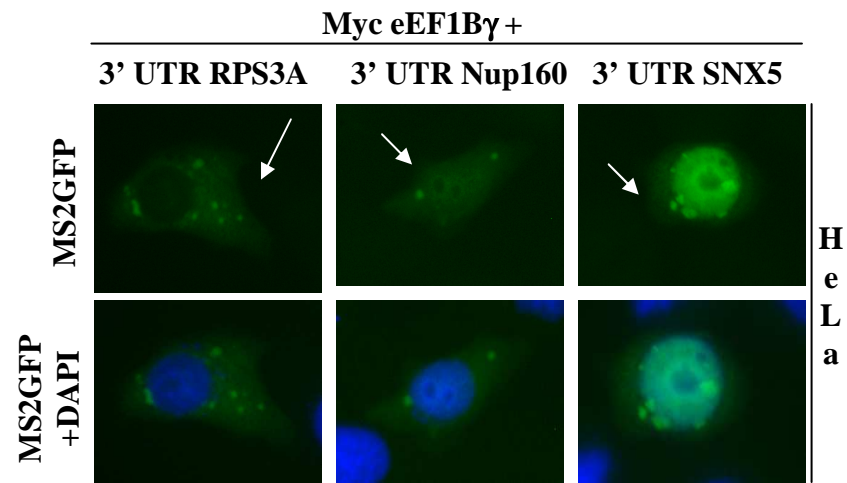**C**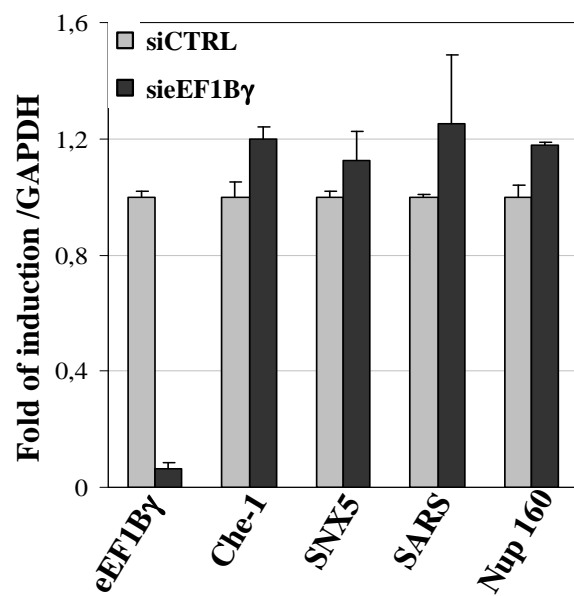**D**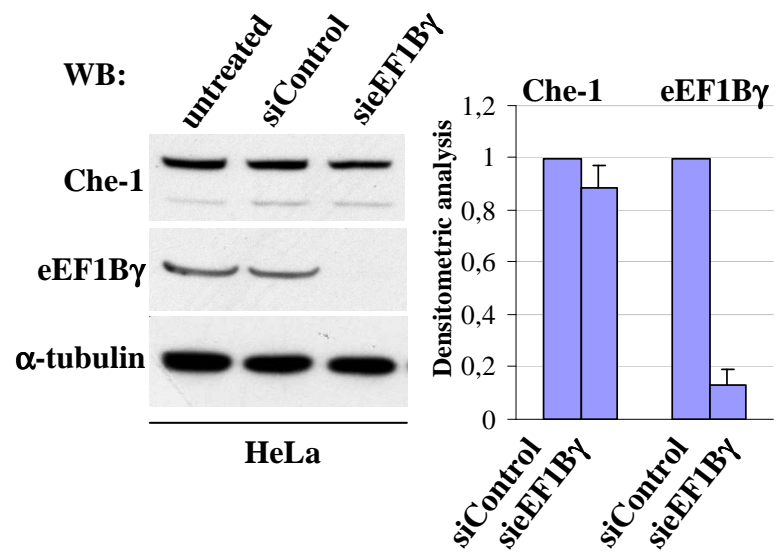

Supplement: Additional file 3: Figure S1. — A. RIP assay output was analyzed by semi-quantitative RT-PCR with specific primers to validate some of mRNAs co-immunoprecipitated with eEF1Bγ and they are listed in Table S2. B. The myc-eEF1Bγ and MS2-GFP fusion proteins were expressed in HeLa cells with the report mRNA carrying both the MS2 binding site and the indicated 3′ UTR. C. RIP assay-eEF1Bγ mRNAs (Additional file 2: Table S2) analyzed by quantitative real time PCR (qPCR) in HeLa whole-cell lysates treated with siRNA as shown. The gene expression ratio between mRNAs and GAPDH are shown as the mean ± SD from three independent experiments performed in triplicate. D. Representative western blot of HeLa whole-cell lysates treated or un-treated with siRNA as shown. The antibodies that were used are indicated. Densitometric analysis represents the mean ± S.D. of 3 independent experiments (right panel). (PDF 208 kb) [file 13046_2016_424_MOESM3_ESM.pdf]

**A**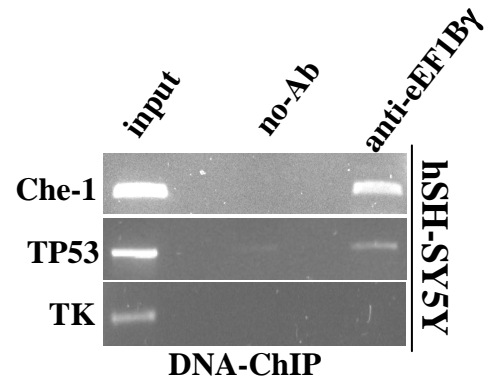**B**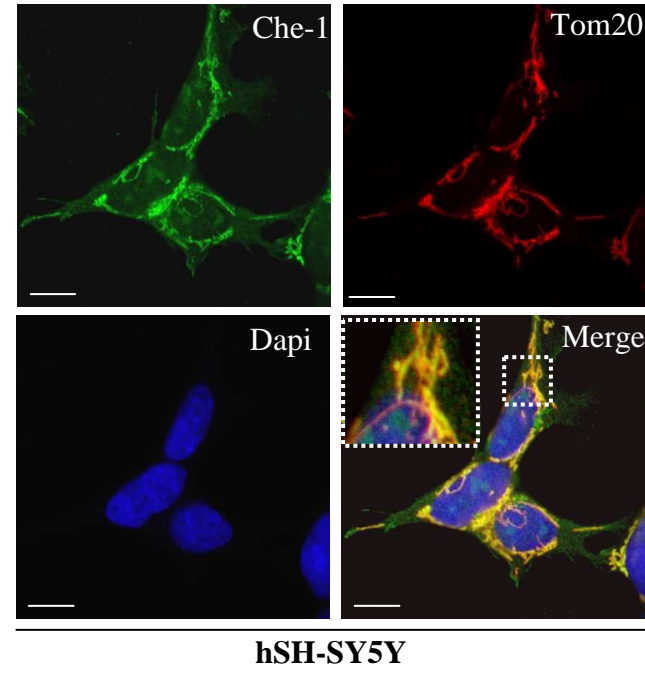**C**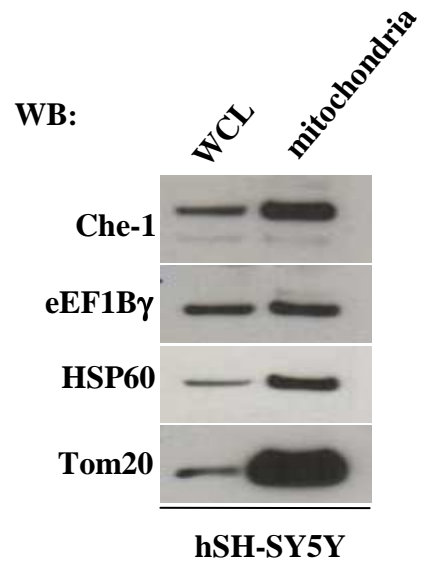**D**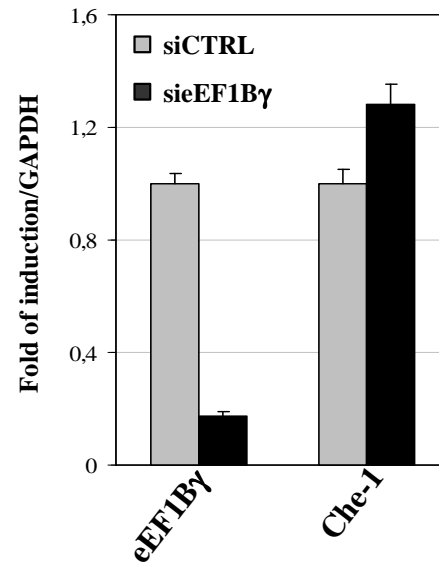**E**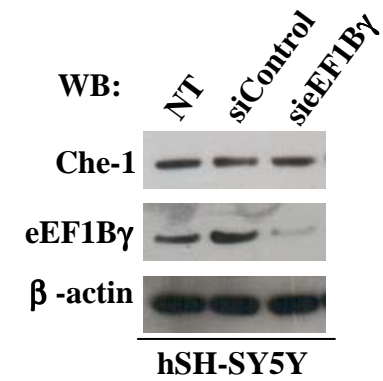

Supplement: Additional file 4: Figure S2. — A. Chromatin immunoprecipitation was performed in hSH-SY5Y cells using anti-eEF1Bγ rabbit polyclonal antibodies or no-Ab as a control. Immunoprecipitates from each sample were analyzed by PCR performed with primers specific for the human Che-1 promoter and for the human TP53 promoter. The thymidine kinase human promoter was amplified as a negative control. A sample representing linear amplification of the total input chromatin (input) was included in the PCR as a control. B. Co-localization of endogenous Che-1, performed with the anti-Che-1 rat polyclonal antibody (green), and the mitochondrial marker Tom20 (red), in hSH-SY5Y cells. Extensive co-localization (yellow) between Che-1 and Tom20 is visualized by the merged-color image. The boxed area represents a high magnification image of co-localization. Nuclei were labeled with DAPI (blue). Scale bars: 10 μm. C. Western blot analysis of hSH-SY5Y whole-cell lysate and mitochondrial enriched fraction. The quality of mitochondrial-enriched fractions was monitored using anti-HSP60 monoclonal antibodies and anti-Tom20 rabbit polyclonal antibodies. D. Quantitative real time PCR (qPCR) analysis of the eEF1Bγ and Che-1 mRNAs in hSH-SY5Y cells (siRNA-Control and siRNA-eEF1Bγ). The gene expression ratio between eEF1Bγ and GAPDH and between Che-1 and GAPDH are shown as the mean ± SD from three independent experiments performed in triplicate. E. Representative Western blot of hSH-SY5Y whole-cell lysates treated or un-treated with siRNA as shown. The antibodies that were used are indicated. (PDF 199 kb) [file 13046_2016_424_MOESM4_ESM.pdf]
